# The contemporary distribution of *Trypanosoma cruzi* infection in humans, alternative hosts and vectors

**DOI:** 10.1038/sdata.2017.50

**Published:** 2017-04-11

**Authors:** Annie J. Browne, Carlos A. Guerra, Renato Vieira Alves, Veruska Maia da Costa, Anne L. Wilson, David M. Pigott, Simon I. Hay, Steve W. Lindsay, Nick Golding, Catherine L. Moyes

**Affiliations:** 1 Oxford Big Data Institute, Li Ka Shing Centre for Health Information and Discovery, University of Oxford, Oxford OX3 7BN, UK; 2 Sanaria Institute for Global Health and Tropical Medicine, Rockville, Maryland 20850, USA; 3 Secretaria de Vigilância em Saúde, Ministério da Saúde, Brasília, Distrito Federal 70058-900, Brasil; 4 School of Biosciences, Durham University, Durham DH1 3LE, UK; 5 Institute for Health Metrics and Evaluation, University of Washington, Seattle, Washington 98121, USA; 6 School of Biosciences, University of Melbourne, Parkville, Victoria 3010, Australia

**Keywords:** Risk factors, Parasitic infection, Epidemiology

## Abstract

Chagas is a potentially fatal chronic disease affecting large numbers of people across the Americas and exported throughout the world through human population movement. It is caused by the *Trypanosoma cruzi* parasite, which is transmitted by triatomine vectors to humans and a wide range of alternative host species. The database described here was compiled to allow the risk of vectorial transmission to humans to be mapped using geospatial models. The database collates all available records, published since 2003, for prevalence and occurrence of infection in humans, vectors and alternative hosts, and links each record to a defined time and location. A total of 16,802 records of infection have been extracted from the published literature and unpublished sources. The resulting database can be used to improve our understanding of the geographic variation in vector infection prevalence and to estimate the risk of vectorial transmission of *T. cruzi* to humans.

## Background & Summary

Chagas disease is caused by the protozoan *Trypanosoma cruzi*. It is endemic across most of Latin America causing high levels of morbidity and mortality. An estimated 10 million people are affected worldwide^1,2^, with approximately 8,000 deaths in 2015 (ref. 3). High levels of geographic variation in the burden of disease have been observed^2,4^ with people in poor, rural areas most at risk^5^. Further spatial variation results from heterogeneity in *T. cruzi* vectors^6,7^, mediated by vector control programmes that are themselves spatially heterogeneous^8,9^. A clear understanding of the spatial variation in *T. cruzi* transmission and infection is required to enable effective implementation of control measures for Chagas disease.

Our understanding of the geographical distribution of *T. cruzi* infection risk is complicated by the multiple routes of transmission, multiple vector species and multiple diverse reservoir species. The parasite is primarily transmitted through the faeces of triatomine bugs contaminating their own bite wounds^9^. Congenital transmission and transmission through blood transfusion and organ donation also occur. Oral transmission is the primary mode by which wild animals are infected due to frequent ingestion of infected triatomines^10^, and outbreaks of orally transmitted *T. cruzi* in humans occur via contaminated food sources, extending the range of vector species that present a risk to humans^11–13^. Vectorial transmission via wounds or contaminated food is the route by which infections enter human populations and control of this transmission route is essential. The database described here was compiled to support analyses of the risk of vectorial transmission to humans.

Understanding variation in vector infection prevalence alone is not enough to understand the risk to humans from vector-related transmission. Several factors affect the relationship between vector and human infection prevalence. For example, vector species vary in their host preferences and this is affected by factors such as local host availability and house condition^14,15^. Additionally, vector control programmes reduce the risk to humans and their efficacy is affected by a range of factors^8,9^. Data on vector infections must therefore be coupled with data on human infections in endemic areas to understand the risk of transmission to humans. Data on human infection is complicated because the parasite can remain in the body for many decades. Acute infections, following a one to two week incubation period, typically last two to three months and during this period parasites can be identified from blood samples but serological testing does not detect infection in the early acute phase^16^. In contrast, parasites are rarely detected from the blood during clinical latency, which can last for decades^17^. Surveys of infection in humans rely on serology so the duration of infection is unknown at the time of testing, making it hard to generate estimates for the location where a subject was originally infected.

In the absence of data on human and vector infections, data on infections in alternative hosts can provide an indication of pathogen presence. Domestic and wild animal species are important reservoirs of *T. cruzi* and are responsible for maintaining parasite populations over long periods of time^14^. Further information can be derived from the known variation in the ability of different host species to sustain and transmit the pathogen^14^ and from their proximity to humans.

**Figure 1:**
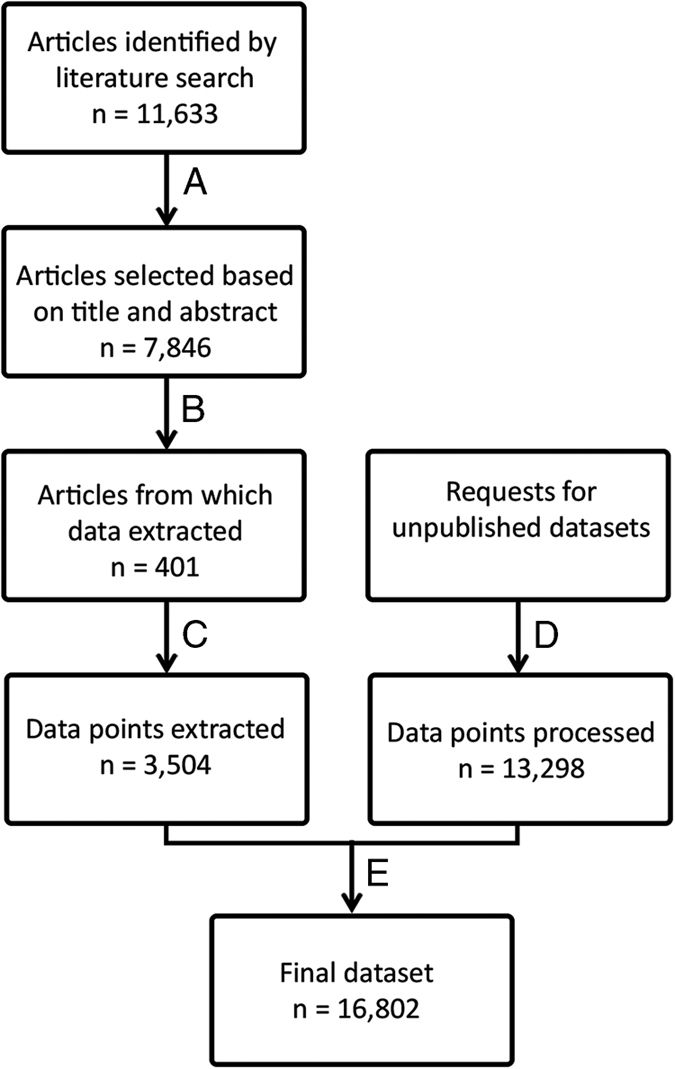
The process from data source identification to data extraction. Steps A-E are described in the Methods section of the main text.

The World Health Organisation’s Global Vector Control Response stresses the importance of conducting surveillance of locally important vectors in addition to monitoring human infection data. Data on infections in human and vector populations thus come from both research studies (usually published) and public health surveillance programmes (often unpublished). We have collated data from both research and surveillance programmes to provide a contemporary database of *T. cruzi* infection in human, vector and alternative host populations across the region. The aim is to provide data for geospatial models that will produce detailed maps for one of Latin America’s most important infectious diseases, providing the basis for more effective targetting of resources. To achieve this we extracted data from the published literature and unpublished sources (Fig. 1). Previously published datasets have focused on one endemic country or one data type^6,18,19^. To our knowledge this is the first database covering the endemic region as a whole and incorporating infection in humans, vectors and alternative host species with each record linked to a defined time and location.

## Methods

### Identifying and selecting data sources

Potential data sources were identified and then selected for data extraction following steps A to E in Fig. 1. The Web of Science bibliographic database was chosen because it incorporates many relevant databases including the SciELO Citation Index from 1997 onwards (provides access to leading journals from Latin America, Portugal, Spain and South Africa), MEDLINE from 1950 onwards (from the U.S. Library of Medicine), the Data Citation Index from 1993 onwards (provides details of datasets in international data depositories), the BIOSIS Citation Index from 1969 onwards (covers pre-clinical, experimental, and animal research) and the Web of Science’s own Core Collection from 1945 onwards. The Web of Science was searched for articles published between 1 January 2003 and 31 December 2015 using the search terms ‘Trypanosoma cruzi’ and ‘Chagas’. This time period was selected to obtain a contemporary dataset while including as many endemic countries as possible. No language restrictions were placed on the search. The initial search yielded 11,633 articles with the term ‘Trypanosoma cruzi’ or the term ‘Chagas’ in the title or abstract or key words.

In the first step of the selection process (A), titles and abstracts were scanned by an individual for any indication that the study measured the occurrence, prevalence or seroprevalence of *T. cruzi* in human communities, or in vector or reservoir populations sampled from the field. Articles that gave no indication that local occurrence or prevalence of infection was measured were discarded during this stage of the process. Typically this excluded studies of, for example, therapeutic options for patients with chronic disease, molecular studies of lab strains of the parasite, mouse model experiments, and so on, unless such studies also incorporated field collected data. This step reduced the number of articles of potential interest to 7,846.

In step B of the process, full text copies were obtained wherever possible and read by an individual. Articles that contained quantitative data on infection prevalence or occurrence in one or more human or vector or alternative host populations, linked to a defined location and time, were retained. If an article contained aggregated data (across multiple locations or multiple collection periods) the authors were contacted and the disaggregated data were requested. Review articles were excluded unless the original data source plus the dates of the original sample collection were provided. This step yielded 401 articles. In addition, if an article mentioned an unpublished dataset or a surveillance programme, the details were noted.

Step C of the process involved the extraction of the data fields listed in the Metadata File from each article identified in step B. First each article was assessed by an individual to determine whether the data met the criteria for infection prevalence data. If so, the relevant fields were extracted and, if not, the fields for infection occurrence were extracted. Seven different data files for infection prevalence and infection occurrence in human, vector and alternative host populations, and for acute infection occurrence in humans, were compiled. The inclusion criteria for each data file, and for the location and time linked to each data point, are detailed below.

Unpublished datasets identified during step B above were requested from the groups managing these data, identified using Google. Step D of the process mirrored step C; each dataset was assessed by an individual to determine whether the data met the criteria for infection prevalence data. If so, the relevant fields were extracted and, if not, the fields for infection occurrence were extracted. Finally, in step E of the process the data extracted from the published articles and from the unpublished datasets were combined into the seven data files listed in the Data Records section.

### Infection prevalence in humans

Studies were included if they sampled a cross-section of the local community at a specific time and place (active surveillance). The only exception to this criterion was any study that sampled a restricted age range within the community; these studies were included together with a record of the age range sampled. Studies of prevalence in a non-representative section of the community, for example pregnant women or hospital patients, were excluded.

If a study gave different prevalence values for different diagnostic tests then the value derived from the number of positives in two or more independent serological tests was taken, i.e. the generally recommended diagnostic. In all instances, the combination of diagnostic tests used to derive the prevalence value given was recorded. The number tested and the number positive were extracted, unless these values were not given in which case the prevalence value was recorded. The age range of those sampled was also recorded. If the study stated that the whole community was tested but did not give an age range, this was recorded as 0 to 99.

### Infection occurrence in humans

Studies within endemic countries that did not meet the criteria for prevalence were included in the occurrence dataset. These included whole community studies if the prevalence value was missing, infections in a community subset, passive surveillance and case reports. Cases specifically attributed to vertical transmission, blood donation or organ donation, and reports of chronic patients, were excluded. Cases identified as an acute infection were recorded separately. For each record, presence/absence was recorded with the number of individuals tested if available. The combination of diagnostic tests used to confirm each presence or absence was also recorded.

### Infections in vectors and alternative hosts

Vectors and reservoir hosts tested were assumed to be a representative sample of the population at that location. The number tested and number positive, or the prevalence, were recorded. If prevalence data were not available then presence/absence was recorded together with the number tested if given. In both instances, the combination of diagnostic or detection tests used was recorded. For each record, the species sampled was recorded using the scientific name, and assigned to one of 19 species groupings.

### Sample collection times and locations

All data were disaggregated to a single collection period and collection site wherever possible. If the published article only contained aggregated data (aggregated over time or over locations), the authors were contacted to request the disaggregated data.

Geographical coordinates were assigned to individual sites that could be located precisely within 5×5 km. If samples from more than one site were pooled before they were tested, all site coordinates were linked to the single record. If an area >25 km^2^ was given by the authors or data source (including hospital catchment areas), this was defined as a polygon. Polygons that matched a country’s administrative divisions were assigned an identifier from the UN Food and Agriculture Organisation’s Global Administrative Unit Layers (GAUL)^20^.

For all sites ⩽5×5 km, geographical coordinates provided in the article or by the data source were converted to decimal degrees. If a map was provided, this was used to assign coordinates to each site. If no coordinates or map were provided, the site name was used together with contextual information such as the district, distance to a border or city and so on, to locate the site in online gazetteers such as Google Maps (http://maps.google.co.uk), Geonames (http://www.geonames.org/) and Open Streep Map (https://www.openstreetmap.org/). If more than one potential site was identified, no coordinates were assigned. Where possible the site coordinates were verified in two separate gazetteers. If the site could not be found in any of the gazetteers then further online searches were used to determine the location. If no precise coordinates could be found then the next highest level geography (e.g., a district polygon) was assigned.

### Data sources

The data source was recorded and up to two data sources were linked to each data point, for example, if prevalence was recorded in a published article but the date of collection was confirmed through a personal communication then both the article and personal communication were linked to the same data point.

### Defining the limits of vectorial transmission of *Trypanosoma cruzi*

The geographical limits of transmission were determined using the published literature and online resources. A mask of areas with no record of vector related human infection was then created using ArcGIS (ESRI 2011. ArcGIS Desktop: Release 10.1. Redlands, CA: Environmental Systems Research Institute).

The continental southern limits of transmission were defined as the Chubut region in Argentina and the Libertador region of Chile. Small numbers of triatomines have been found in Chubut^21^ but all tested negative for *T cruzi* whilst infections have been reported in the seven northern regions of Chile but not in the south^22^. Maps from the Pan American Health Organisation (PAHO) and the International Association for Medical Assistance to Travellers (IAMAT) provide clear advice for no risk of vector-related *T. cruzi* transmission south of these regions (https://www.iamat.org/risks/chagas-disease, http://www.paho.org/salud-en-las-americas-2012).

The northern limits of Chagas are less clearly defined. Autochthonous cases arising from vectorial transmission have been reported in humans in Louisiana and Texas^23,24^ and in mammals and triatomines across the southern USA and as far north as Tennessee^23–41^. Based on these observations and a map of states reporting triatomines (www.cdc.gov/parasites/chagas/gen_info/vectors), the northern limit was drawn from the north of California to the north of Pennsylvania.

The World Health Organisation (WHO) and the Centers of Disease and Control and Prevention (CDC) state that vector-related transmission does not occur in the Caribbean (www.who.int/mediacentre/factsheets/fs340; www.cdc.gov/parasites/chagas/gen_info/vectors). The only Caribbean country reporting Chagas cases is Grenada^42,43^ therefore all other Caribbean countries were deemed not at risk. The Galapagos Islands were deemed not at risk because studies have identified an absence of triatomine vectors^44^ and serological testing of dogs and cats showed no evidence of infection^45^. Uruguay is the only continental Latin American country to have interrupted vector-related transmission of *T. cru*zi (www1.paho.org/english/ad/dpc/cd/incosur.htm). Since control of transmission in 1998, there have been no reports in Uruguay and therefore it was also classified as not at risk.

## Data Records

The full workflow and numbers of articles contributing to the final dataset is given in Fig. 1. A total of 16,802 data records were extracted and input into seven data files, stored in the Dryad Digital Repository (Data Citation 1).

The seven individual data files within Data Citation 1 are (1) prevalence of infection in humans, (2) occurrence of acute infection in humans, (3) occurrence of infection in humans, (4) prevalence of infection in vectors, (5) occurrence of infection in vectors, (6) prevalence of infection in alternative hosts, and (7) occurrence of infection in alternative hosts. Each data file contains individual records linked to a defined time and location. Locations are defined as either points (an area ⩾5×5 km, assigned geographical coordinates in decimal degrees), or polygons (areas >5×5 km with defined boundaries). Times are defined as the start month and year, and the end month and year, for each sample collected. The total number of records is given in Table 1.

**Figure 2:**
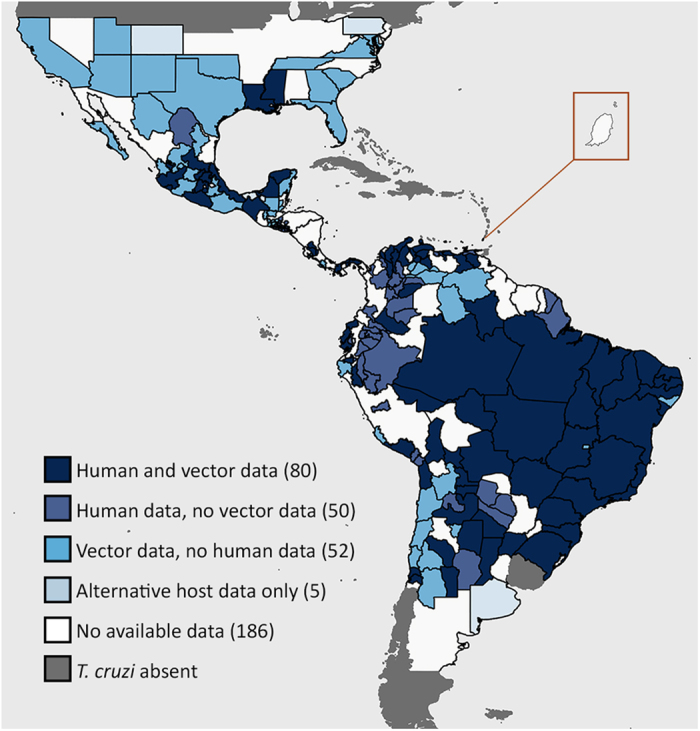
Data availability by first level administrative division. For each first order administrative division (typically called a state or department) the types of data available are shown and the total number of administrative divisions with data is given in parentheses. The combination of human and vector infection data is prioritised and the availability of alternative host infection data is only shown if no other data types are available. An enlarged view of the island nation Grenada is shown.

All data records were linked to sub-national geographic locations. Data were obtained for 20 of the countries where Chagas is endemic but no data records were identified from Suriname, Guyana or Grenada. Figure 2 highlights states/departments with no available data. Despite some obvious gaps, we provide data on human and/or vector infections for most of the endemic region. At the northern and southern fringes of the regions some areas only have data for alternative hosts. Areas with no publicly available data at all (published since 2003) can be seen throughout the endemic region.

Within the data file for prevalence of infection in vectors, values are recorded for a total of 59 triatomine species. The mean prevalence of infection in the five most commonly sampled species is given in Table 2. These species are all found in Brazil and their status as the most widely tested species largely reflects the high number of locations and volume of data from Brazil. It is interesting to note that although the database has only 162 prevalence records for *T. infestans* populations, a large number of specimens have been tested (11,994, with a mean infection prevalence of 35.4%), reflecting the importance of this vector within its restricted range^46^. Many other species are locally important with higher infection prevalences than those found for the most widely sampled species.

The data files for occurrence and prevalence of infection in alternative hosts encompass 177 species including rodents, bats, non-human primates, other wild mammals, marsupials, domestic mammals and livestock. The mean prevalence of infection in the most sampled species is given in Table 3 and it can be seen that the most widely tested alternative host is the domestic dog, *Canis familiaris*, with over 250 records of infection prevalence.

The aim of this work is to provide contemporary records of infections and only published articles from 2003 onwards were included. Studies of historical trends in the location and prevalence of infection will need to expand the current dataset further by extracting data from older publications. The current data compilation exercise ended in 2016 and, as time passes, increasing amounts of more recent data will also become available so the literature should be monitored for new records by any groups wishing to update the current dataset. Finally, the dataset presented here may not be complete. Potential records of interest may have been missed during each step of the process outlined in Fig. 1 and there are likely to be unpublished datasets that were not identified or obtained. There is no comprehensive dataset in existence that would allow an assessment of completeness of the current database, however, a georeferenced database of infections in North America has been compiled that contains 669 records from 1936 to 2014 (ref. 6). Here we collated 341 infection records for Mexico and the USA (excluding absence records) from articles published from 2003 to 2015. The average number of records per year is higher in the current dataset, possibly because more data have been generated in more recent years.

## Technical Validation

Each data record was extracted by one individual and checked by a second person to ensure accuracy. All site coordinates linked to data records were checked using GIS software (ArcGIS and QGIS) to ensure they fell on land (as defined by GAUL) and in the right country. All point and polygon locations for each data source were plotted to ensure they matched the sampling design described by the data source.

All other fields were checked to confirm that each value fell within the expected range and to identify any missing data. If any information was unclear then authors were contacted for confirmation.

Each full data file was checked to ensure that study records had not been duplicated. The data file for each vector species was also plotted and any outliers geographically distant from the main distribution were investigated to ensure they had been correctly extracted and fell within the species range.

## Usage Notes

**Figure 3:**
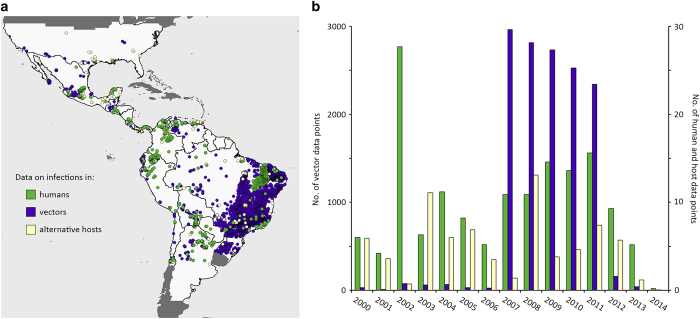
Spatial and temporal clustering in the dataset. (**a**) The map shows the spatial distribution of the human, vector and alternative host populations sampled. (**b**) The plot shows the distribution of the data across years for infections in humans, vectors and hosts.

This dataset does not represent a systematic survey across the Chagas endemic region using a single consistent method. It is a collation of observational data containing inherent biases that should be addressed when the data are used in geospatial models. Figure 3 shows that the distribution of data is not uniform in either time or space and this is in part due to sampling bias. The choice of study locations is biased with a preference for areas of known high infection prevalence or areas linked to risk factors such as poor housing in rural localities^5^. Analysis of these data should therefore make use of methods that have been developed to account for such sampling biases^47,48^. The full dataset provides the information needed to assess these data appropriately so that users can also take account of potential confounders such as the combination of diagnostics used, species tested, and age of the subjects sampled. Other important factors contributing to variation in the results recorded, such as the sample size for each record and the number of records for a particular location, are also held within the dataset. This allows modellers to incorporate the strength of evidence associated with each record of disease occurrence or prevalence, for example taking account of the often highly stochastic nature of empirical infection prevalence estimates, especially in vectors.

This database was collated to support analyses that map the risk of vectorial transmission of *T. cruzi* to humans. The inclusion of data on the location and time of each sample collection means that this dataset can be used in combination with spatiotemporal environmental, demographic and socioeconomic covariates to model the risk of human infection at fine spatial resolution across the Americas. Ultimately we hope that these data will be used to improve knowledge of the geographical extent of endemic Chagas disease and disease burden. Understanding the relationship between the prevalence of *T. cruzi* in vectors and the risk of infection to humans will help inform appropriate control measures and highlight areas where resources are required.

## Additional Information

**How to cite this article:** Browne, A. J. *et al.* The contemporary distribution of *Trypanosoma cruzi* infection in humans, alternative hosts and vectors. *Sci. Data* 4:170050 doi: 10.1038/sdata.2017.50 (2017).

**Publisher’s note:** Springer Nature remains neutral with regard to jurisdictional claims in published maps and institutional affiliations.

## Supplementary Material



## Figures and Tables

**Table 1:** Data volumes for each file within the dataset.

**Data file**	**Number of records**	**Number of locations**	**Number of countries**
Prevalence in humans	1,012	607	15
Acute infections in humans	497	216	8
Occurrence in humans (excluding prevalence data and confirmed acute infections)	328	128	12
Prevalence in vector species	13,798	2,755	15
Occurrence in vector species (excluding prevalence data)	276	220	10
Prevalence in alternative host species	858	338	13
Occurrence in alternative host species (excluding prevalence data)	33	21	7
A data record is defined as a prevalence value or presence/absence for a defined location and collection period derived from a unique study. The number of locations refers to the number of unique locations with infection data.			

**Table 2:** Prevalence of *T. cruzi* infection in the most sampled vector species.

**Vector species**	**Number of records**	**Mean prevalence (%)**
*Triatoma sordida*	2,502 (342,791)	1.0 (0–100)
*Triatoma pseudomaculata*	2,286 (118,064)	4.6 (0–100)
*Panstrongylus megistus*	1,951 (20,897)	10.2 (0–100)
*Triatoma brasiliensis*	1,795 (104,851)	4.6 (0–100)
*Rhodnius neglectus*	1,371 (9,804)	3.7 (0–100)
A data record is defined as a prevalence value for a defined location and collection period derived from a unique study. The total number of specimens tested is given in parentheses after the number of records, and the full range of prevalence values is given in parentheses after the mean prevalence value.		

**Table 3:** Prevalence of *T. cruzi* infection in the most sampled alternative host species.

**Host species**	**Species grouping**	**Number of records**	**Mean prevalence (%)**
*Rattus rattus* (black rat)	Rodent	22 (171)	26 (0–100)
*Sigmodon hispidus* (cotton rat)	Rodent	11 (316)	17.7 (0–50)
*Octodon degus* (degu)	Rodent	10 (861)	44 (13.3–70.4)
*Carollia perspicillata* (Seba’s short-tailed bat)	Bat	8 (150)	25.9 (0–54.5)
*Leontopithecus rosalia* (golden lion tamarin)	Non-human primate	5 (607)	33.9 (25.1–43.7)
*Nasua nasua* (South American coati)	Other wild mammal	11 (343)	52 (0–100)
*Procyon lotor* (raccoon)	Other wild mammal	11 (302)	11.3 (0–55)
*Nasua narica* (white-nosed coati)	Other wild mammal	10 (126)	20.2 (0–93.3)
*Didelphis marsupialis* (common opossum)	Marsupial	20 (111)	48.9 (0–100)
*Didelphis albiventris* (white-eared opossum)	Marsupial	18 (391)	34.4 (0–88.9)
*Monodelphis domestica* (gray short-tailed opossum)	Marsupial	12 (227)	19 (0–100)
*Canis familiaris* (domestic dog)	Domestic mammals	253 (11,506)	24.6 (0–100)
*Felis catus* (domestic cat)	Domestic mammals	25 (832)	16.3 (0–62.1)
*Mus musculus* (house mouse)	Domestic mammals	19 (231)	5.5 (0–20)
*Sus scrofa domesticus* (domestic pig)	Livestock	11 (142)	29.7 (0–100)
*Ovis aries* (sheep)	Livestock	4 (308)	1.5 (0–3.9)
A data record is defined as a prevalence value for a defined location and collection period derived from a unique study. The total number of specimens tested is given in parentheses after the number of records, and the full range of prevalence values is given in parentheses after the mean prevalence value.			
