# Corrigendum: The contemporary distribution of *Trypanosoma cruzi* infection in humans, alternative hosts and vectors

**DOI:** 10.1038/sdata.2017.71

**Published:** 2017-05-30

**Authors:** Annie J. Browne, Carlos A. Guerra, Renato Vieira Alves, Veruska Maia da Costa, Anne L. Wilson, David M. Pigott, Simon I. Hay, Steve W. Lindsay, Nick Golding, Catherine L. Moyes

**Keywords:** Risk factors, Parasitic infection, Epidemiology

*Scientific Data* 4:170050 doi: 10.1038/sdata.2017.50 (2017); Published 11 April 2017; Updated 30 May 2017

The financial support for this Data Descriptor was not fully acknowledged. The Acknowledgements should have included the following: S.I.H. is funded by a Senior Research Fellowship from the Wellcome Trust [095066].

